# Mind the Step: An Artificial Intelligence-Based Monitoring Platform for Animal Welfare †

**DOI:** 10.3390/s24248042

**Published:** 2024-12-17

**Authors:** Andrea Michielon, Paolo Litta, Francesca Bonelli, Gregorio Don, Stefano Farisè, Diana Giannuzzi, Marco Milanesi, Daniele Pietrucci, Angelica Vezzoli, Alessio Cecchinato, Giovanni Chillemi, Luigi Gallo, Marcello Mele, Cesare Furlanello

**Affiliations:** 1 Orobix Life, 24121 Bergamo, Italy; stefano.farise@orobix.com (S.F.); angelica.vezzoli@orobix.life (A.V.); cesare.furlanello@light-center.it (C.F.); 2 Antares Vision, 25039 Travagliato, Italy; 3 Department of Veterinary Sciences, University of Pisa, 56124 Pisa, Italy; francesca.bonelli@unipi.it; 4 Department of Agronomy, Animals, Food, Natural Resources, and Environment—DAFNAE, University of Padua, 35020 Legnaro, Italy; gregorio.don@unipd.it (G.D.); diana.giannuzzi@unipd.it (D.G.); alessio.cecchinato@unipd.it (A.C.); luigi.gallo@unipd.it (L.G.); 5 Department for Innovation in Biological, Agri-Food, and Forestry Systems—DIBAF, University of Tuscia, 01100 Viterbo, Italy; marco.milanesi@unitus.it (M.M.); daniele.pietrucci@unitus.it (D.P.); gchillemi@unitus.it (G.C.); 6 Department of Agriculture, Food and Environment—DAFE, Università di Pisa, 56124 Pisa, Italy; marcello.mele@unipi.it; 7 LIGHT Center Brescia, 25123 Brescia, Italy

**Keywords:** AI/ML, precision livestock farming, locomotion score, body condition score, behavioral analysis

## Abstract

We present an artificial intelligence (AI)-enhanced monitoring framework designed to assist personnel in evaluating and maintaining animal welfare using a modular architecture. This framework integrates multiple deep learning models to automatically compute metrics relevant to assessing animal well-being. Using deep learning for AI-based vision adapted from industrial applications and human behavioral analysis, the framework includes modules for markerless animal identification and health status assessment (e.g., locomotion score and body condition score). Methods for behavioral analysis are also included to evaluate how nutritional and rearing conditions impact behaviors. These models are initially trained on public datasets and then fine-tuned on original data. We demonstrate the approach through two use cases: a health monitoring system for dairy cattle and a piglet behavior analysis system. The results indicate that scalable deep learning and edge computing solutions can support precision livestock farming by automating welfare assessments and enabling timely, data-driven interventions.

## 1. Introduction

The growing global demand for animal products has driven the expansion of large-scale intensive farming, particularly in the meat and dairy sectors. This trend poses significant challenges related to resource exploitation, environmental sustainability, and animal welfare. As a result, there is an urgent need for innovative solutions that balance productivity with ethical practices. Precision livestock farming (PLF) has emerged as a pillar in addressing these challenges. By enhancing the efficiency and sustainability of livestock production, PLF utilizes advanced monitoring and management techniques [1]. Technologies such as sensors, cameras, and artificial intelligence (AI) collect and analyze real-time data on animal health, behavior, and welfare [2]. This data-driven approach allows for targeted interventions tailored to individual animals, improving productivity while ensuring compliance with ethical standards. In Italy, the national AGRITECH center is leading the charge in developing technologies for sustainable agriculture as part of the National Recovery and Resilience Plan. With a dedicated division (Spoke 5) focused on enhancing animal welfare and minimizing the environmental impact of livestock systems, AGRITECH is exploring less intensive alternatives within PLF to find a balance between productivity and sustainability. PLF plays an important role in monitoring essential operations such as precision feeding, milking, and climate control. This not only helps adhere to national and international animal welfare regulations but also provides objective quality control. A significant challenge in adopting PLF is the limited availability of trained personnel to oversee the collection of commonly used veterinary metrics of interest, such as the body condition score and locomotion score. Here, AI becomes a crucial enabler of sustainable PLF by automatically and continuously monitoring each animal’s status and tracking its evolution over time while maintaining ease of interpretation, as it provides both visual and numerical assessments. This becomes a crucial tool to alert a hypothetical farmer using the system to anomalous animal behaviors, such as metabolic disorders or lameness, ahead of time. An automated and continuous quality control system is deployable across various farm scales, thanks to the flexibility provided by AI itself. AGRITECH is integrating AI and machine learning (ML) modules into existing PLF systems to extract vital data and support informed decision-making.

A second challenge is to bring AI models into edge computing implementations, that is, to operate independently of cloud connections, making them suitable for deployment in the field. This work evaluates the feasibility of operating the AI animal welfare modules effectively in barn structures, thus ensuring a practical application of AI across diverse farming environments [3]. The two AI-based systems presented in this work demonstrate a novel framework for precision livestock farming, combining modular, vision-based architectures with adaptable designs for species-specific welfare indicators. The cattle-focused system automates health metrics like locomotion and body condition scores, while the pig-focused system captures behavior-driven welfare cues. These distinct designs share a common hardware and architectural foundation, underscoring the scalability and versatility of the approach across diverse farming contexts. In this work, we demonstrate that close-to-real-time performance is achievable with different types of hardware (GPUs), particularly when considering the block-like structure of systems, which is easily parallelizable. This approach, combined with the regular barn management cycle—which provides large time windows with minimal or sporadic events (between milking for system 1 and at night for system 2)—further enhances efficiency. Furthermore, digital analyses and their resulting metrics, automatically estimated through vision AI-based methods, are key enablers for digital spaces dedicated to sustainable agriculture, such as the METRIQA platform developed by Chessa and colleagues [4] within AGRITECH. METRIQA implements a data federation approach, based on the Data Space architecture [5]. It aggregates data from a wide array of sources, including camera systems, environmental sensors, and wearable devices, enabling continuous monitoring of livestock farms. This work can thus be integrated into METRIQA and similar platforms. Vision-based AI solutions can also contribute to automating evaluation processes based on other sensors, for example, in outdoor grazing. Vision-based AI systems that capture activity and pathophysiological features can be translated into actionable PLF scores based on well-designed observational setups [6,7]. These solutions can now also be based on public dataset resources; our approach is indeed bootstrapped on videos from public repositories [8,9], as described in Section 2.1.3. Moreover, they can also serve as trainers for other sensor-enabled monitoring applications. The automatic evaluation of features distilled by AI classifiers from video streams can indeed play a key role in the calibration of wearable sensors (e.g., collars), providing continuous labeling, as implemented for the calibration of low-cost Inertial Measurement Units (IMUs) for the detection of human or robot activity features [10]. More specifically, AI-based models can be instrumental in extracting features from source videos and supporting the training of professionals. Considering these prospective applications, we introduce our two first AI-based systems that have reached a continuous evaluation testbed and discuss their potential for edge computing implementation.

## 2. Materials and Methods

### 2.1. System 1—Health Status Assessment

#### 2.1.1. Setup and Data

**Figure 1:**
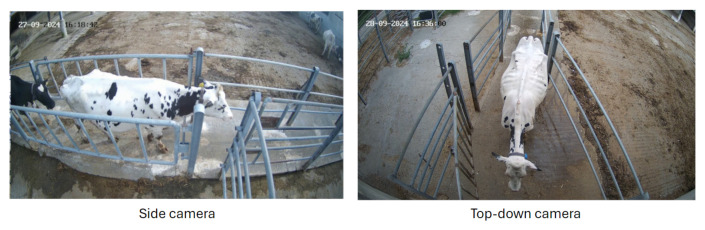
The dairy cattle two-camera setup. The side-view camera is used for the estimation of pose and locomotion features, while the top-view camera supports markerless identification.

From a hardware standpoint, the setup consists of two standard security cameras, both set at a resolution of 3200 by 1800 pixels. The cameras are strategically placed to monitor the corridor used to reintroduce cattle from the milking parlor back into the barn (Figure 1). The side camera acquires short clips, triggered by motion detection within the region of interest (ROI). When an animal walking across the frame reaches its rightmost part, a timed snapshot from the top-down camera is automatically captured. The side camera captures clips at a frequency of 20 frames per second. The top-down camera acquires a single snapshot of the whole body of the animal. The system is currently applied to acquisition and validation campaigns monitoring 37 Holstein-Friesian dairy cows that are regularly milked twice a day. The acquisition and computation are handled by a standard workstation (HP Inc., Palo Alto, CA, USA) deployed in the milking parlor office, approximately 15 m from the entrance of the corridor. This setup represents an edge computing solution, with the computer being an HP Z1 Tower G9 R, configured with an NVIDIA GeForce RTX 3060 GPU. Additionally, the performance of the models has been tested on various hardware setups, including different tiers of GPUs, such as the NVIDIA GeForce RTX 2060 and the NVIDIA H100.

#### 2.1.2. Cattle Detection and Tracking

**Figure 2:**
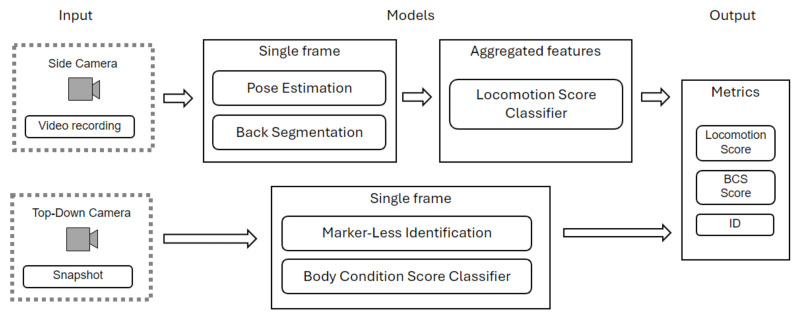
Architecture of the AI health status assessment system.

The overall framework for health status assessment (Figure 2) is based on the two-camera setup with two complementary pipelines. Both the side and the top-down pipelines are initialized by a specialized object detection YOLOv8 neural network [11]. In the case of the side view, the model is also utilized for tracking the animal within the input frame. The cattle are thus identified and tracked, and all irrelevant background information is removed. Our YOLOv8 model has been pre-trained using the COCO (Common Objects in Context) dataset [8], which includes a class for cow detection among its 80 classes. Further, we fine-tuned the model on a specialized cattle dataset, which we curated to include images with both lateral and bird’s-eye views. This dataset comprises a total of 13,629 images, combined from three datasets: OpenCows2020 [12], NWAFU [9], and CVB [13]. Finally, we collected 2418 images in the field at CIRAA, CERZOO, and private barns in the Brescia area. The data were shuffled and partitioned into training, validation, and test sets following a 70-20-10 ratio. High object detection accuracy (mAP50: mean average precision at 50% intersection over union) was obtained on the test set: mAP50=0.978.

#### 2.1.3. Pose Estimation and Locomotion Features

**Figure 3:**
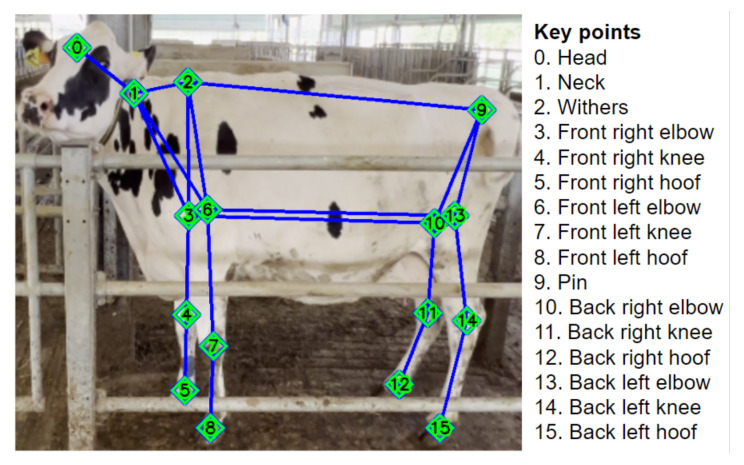
Ordered list of pose key points and an annotation example over a training set image.

For the side camera pipeline, each clip is processed frame by frame, and the object detection and tracking models are applied. Each extracted sub-image is then fed into a posture estimation model, the YOLOv8-pose [11] model trained to estimate the posture of the cattle in the frame as a set of 16 key points (Figure 3), with the same format proposed for multi-branch network modeling [9]. The YOLOv8-pose model also underwent pre-training on the COCO key points dataset [8], followed by fine-tuning based on a dataset of 3288 annotated cattle images from the NWAFU dataset [9], and an additional 400 in-the-field acquisitions, including annotation and the application of a custom augmentation function to enhance the model’s robustness to real-world conditions. An mAP50=0.972 was obtained for the pose estimation task on the test set.

The key-point representation is utilized to extract a set of raw features, including step sizes, the relative head position, and each leg’s hoof shift from its elbow (Figure 3). Moreover, using the withers and the pin key points, an ROI is extracted for the silhouette of the cattle’s back. This ROI is fed into a convolutional neural network (CNN) segmentation model to robustly extract its contour edge. The segmentation model was trained on sub-images of the back area from 1218 images chosen from the cattle pose estimation dataset, achieving an mAP50=0.951 on the test set. These edge points are used to define the back straightness score, which is computed as the normalized error between the back edge and its linear approximation. In the normalization, 1 represents a straight back edge, while 0 represents a very pronounced arching of the back. The set of features extracted from the module is listed in Table 1.

#### 2.1.4. Locomotion Score Classifier

**Figure 4:**
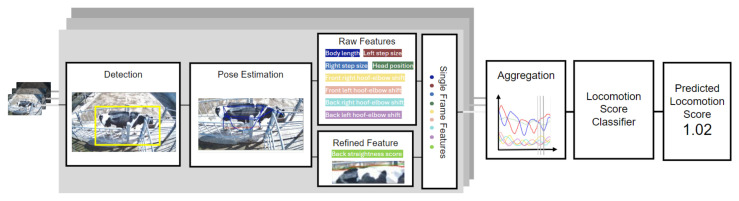
Structure of the locomotion score classifier. The process starts with a clip, divided into a sequence of frames (**left**), which are then processed into a sequence of meaningful features (**middle**). These features are evaluated by the classifier, resulting in a locomotion score prediction (**right**).

The locomotion score (LS) is a visually assessed metric that evaluates the gait of dairy cattle on a scale from 1 (normal) to 5 (severely lame). This metric helps in the early identification of lameness, optimizing hoof care, and improving overall animal welfare and productivity. To compute this metric automatically, we used the 8 locomotion features (Table 1) from the lateral camera video feed, obtaining a sequence of locomotion features over time. A rolling window over this combined signal was used as input to a long short-term memory (LSTM) network to estimate an LS from 1 to 5. The final locomotion score for the clip was computed as the mean of the estimated scores for all the windows in the score. This LSTM-based classifier was trained using the outlined pose estimation pipeline on a dataset of 74 video clips acquired during an on-field campaign at the CIRAA barn, with locomotion scores annotated by trained personnel on-site following the methodologies and scoring system described in [14]. Training was extended with clips from publicly available resources, primarily video lessons used for LS assessment and training. On the test set, the system (Figure 4) achieved an accuracy of 94% for the single-window classification and an $R^{2}=0.96$ for the whole clip length.

#### 2.1.5. Body Condition Score Classifier

**Figure 5:**
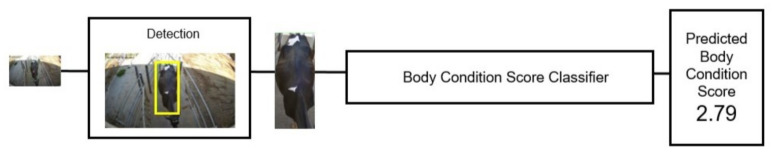
Structure of the body condition score classifier. The process begins with a snapshot, from which the subject is extracted using a detection model. The extracted subject is then evaluated by the body condition score classifier, producing an estimated BCS value.

**Figure 6:**
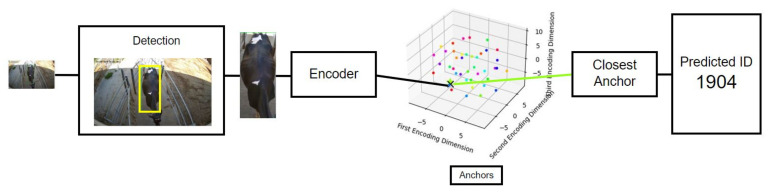
Structure of the markerless identification algorithm. The process starts with a snapshot, where the subject is extracted using a detection model (**left**). The extracted subject is then encoded into a latent space via an encoder network (**middle**). Finally, the closest anchor point is identified using the Euclidean distance (**right**).

The top-down camera pipeline is based on a single snapshot of the cow after ID detection by the fine-tuned YOLOv8. The detection model also crops the frame to the sub-image containing the cow’s body, which is utilized as input for two different models working in parallel: a BCS classifier (Figure 5) and a markerless identification network (Figure 6). The BCS is a standard visually assessed metric that quantifies dairy cattle’s fat reserves on a scale from 1 (emaciated) to 5 (obese). The BCS helps optimize feeding, monitor health, and improve reproductive performance and milk production. A simple ResNet-based CNN was trained as a regression to estimate the score of the cow from 1 to 5. The training dataset for the BCS task was collected during an initial acquisition campaign at CIRAA using our experimental setup. The 37 individuals were observed while moving along the corridor, associating 60 snapshots per animal with its ID and BCS, annotated by trained personnel. The methodologies and scoring system used to evaluate the BCS are described in [15]. An $R^{2}=0.83$ was achieved on the test set.

#### 2.1.6. Markerless Identification

The markerless identification model (Figure 6) employs a TripletNet, an architecture commonly used for face recognition due to its ability to learn robust discriminative features while remaining compact [16]. Our TripletNet consists of two main components: an encoder network and an anchor dictionary. The encoder network is a shallow CNN trained to project the black-and-white pattern of the cattle’s coat into a 3D latent space, forcing similar input images to be closer within the latent space. Images of the same subject are clustered together, and the anchor dictionary maps latent space partitions to their corresponding ID codes. The dataset used to train the BCS classifier was also used to generate triplets by associating each image of a given ID with positive samples (images of the same ID) and negative samples (images from different IDs). The anchor dictionary is then constructed by computing, for each ID, the center of mass of the training encodings. During inference, an input image is associated with the ID in the dictionary corresponding to the anchor closest to the encoded version of the image. The accuracy achieved on the test set was 98%. Notably, the compact nature of the encoder ensures very low inference times. A confidence threshold can then be established based on the maximum allowable distance between an encoded point and an anchor in the dictionary. If no anchor falls within this threshold, the identification fails, indicating either an unsuitable acquisition of a known cow or the presence of a new cow in the system. Finally, the model can be dynamically adjusted as the cattle population grows, eliminating the need for formal retraining. This allows for seamless updates and continuous adaptation of the model to the evolving dataset.

### 2.2. System 2—Behavioral Analysis

#### 2.2.1. Setup and Data

Video recordings were collected at the experimental farm of the University of Padua. The setup was organized in 8 pens, each equipped with an automatic feeder, two drinkers, and 3 enrichment objects (hay dispenser, pole, and ball). Each pig was uniquely identified by a colored ear tag or a livestock mark. An AXIS P3267-LVE Dome camera was installed in each pen, recording at a frame rate of 15 frames per second (fps) and a resolution of 2592×1944 pixels. Recordings were collected from two separate batches: the first batch of 88 pigs (11 per pen), observed from July 2023 to December 2023, was used to train the AI models and tune the system parameters. Below, we provide a breakdown of the number of frames extracted and annotated for each task. It is important to note that 70% of these frames were allocated to the training set, 20% were reserved for the validation set, and the remaining 10% were used for testing. The mAP50 values, presented below for each task, were estimated based on the testing subset. The second batch of 96 pigs (12 per pen) was observed from March 2024 to September 2024. The first two weeks of observations from the second batch were designated as additional testing datasets to evaluate performance and validate accuracy.

#### 2.2.2. Identification and Monitoring Models

**Figure 7:**
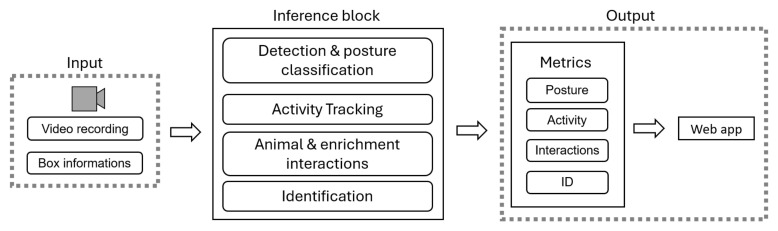
Outline of the pipeline for behavioral analysis.

For the analysis of the video recordings, our pig monitoring system (Figure 7) integrates all the information related to the pen, including the pig IDs, ear tags, livestock marks, and coordinates of fixed objects (i.e., feeder, drinker, hay, pole) inside the pen. All these data are passed to the inference block, where a series of cascading YOLOv8 models [11] are used to extract the behavioral metrics of interest, such as the time spent standing or lying down, distance traveled, and interactions. These metrics are then linked to individual pigs, recognized through ear tag-based or livestock mark-based identification, and visualized using a web app.

#### 2.2.3. Pig Detection, Classification, and Tracking

To ensure accurate detection of multiple animals and maintain high computational speed, a YOLOv8 [11] model is used for pig detection. The model was trained on a dataset containing 433 images (4763 manually annotated instances), defining two different classes (standing vs. lying down) to also achieve pig posture classification. This model achieved an overall mAP50=0.984. The ByteTrack [17] algorithm is used to track the animals frame by frame, ensuring that each animal retains its initial ID. Additionally, a history of coordinates for each pig is maintained to support ByteTrack in case of occlusions caused by the overlap of two or more animals.

#### 2.2.4. Activity Tracking and Body-Part Segmentation

**Figure 8:**
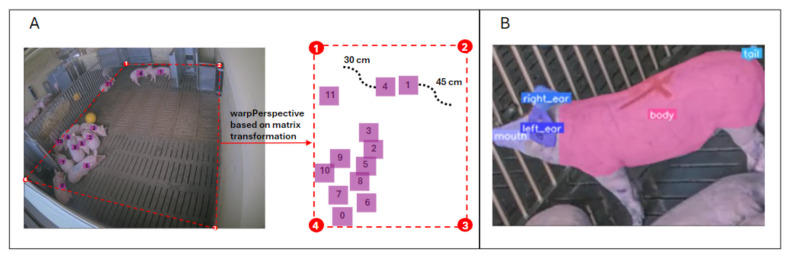
(**A**) Example of how perspective transformation is applied to map pixel coordinates to real-world coordinates, aimed at achieving more precise quantification and characterization of traveled distances. (**B**) Example of body-part segmentation, with the five segmentation classes (mouth, right ear, left ear, body, and tail) highlighted in different colors and labeled accordingly.

Tracking pig activity and estimating the distance traveled is made possible by combining the output of the detection model with perspective transformation (Figure 8). The pixel coordinates of the four corners of the pen are used to map the original image to the real dimensions of the box in centimeters (380×580 cm). The perspective transformation matrix based on these points is then applied to the coordinates of each pig’s centroid. Using this approach, it is possible to quantify the movement of pigs, for instance, to highlight abnormal behaviors such as repeated circular movements. The system continuously estimates the Euclidean distance traveled by standing pigs. A YOLOv8 instance segmentation model is used to identify each pig’s ROI, including its body, tail, mouth, and ears (Figure 8). This model was trained on a dataset of 625 images and achieved an overall mAP50=0.922. This task is key both for the identification of individual animals and for classifying interactions between them at a fine level.

#### 2.2.5. Interactions

**Figure 9:**
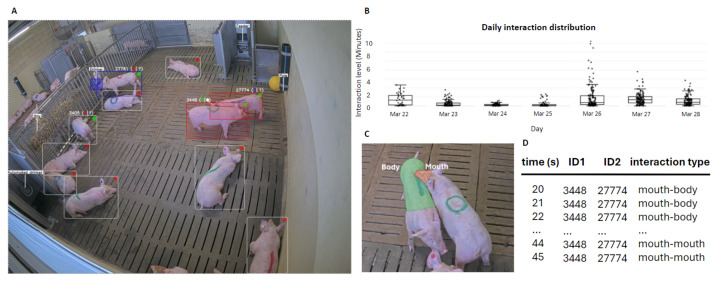
The pig interactions pipeline. (**A**) Inference annotations; (**B**) Daily interaction distribution for Week 1 in Pen 1: total interaction time (minutes) per pig per hour; (**C**) Classification of interactions based on body-part segmentation; (**D**) Example of quantitative analysis of an interaction.

Interactions are computed at both the animal-to-animal and animal-to-enrichment levels, as exemplified in Figure 9. Contact between two animals is first determined by the percentage of overlap between their bounding boxes. Interactions are then evaluated by comparing the animals’ centroid positions and assessing the structural similarity index (SSIM) between the previous and the current frames. If the two animals in contact are moving or changing posture, the interaction is confirmed. Additionally, the system attempts to estimate fine-grained interaction by computing the intersection over union (IoU) between the different body parts of the two animals involved in the interaction. Interactions with enrichment are detected simply by assessing the overlap between the animal and the enrichment mask, or its movement, in the specific case of the ball.

#### 2.2.6. Identification

**Figure 10:**
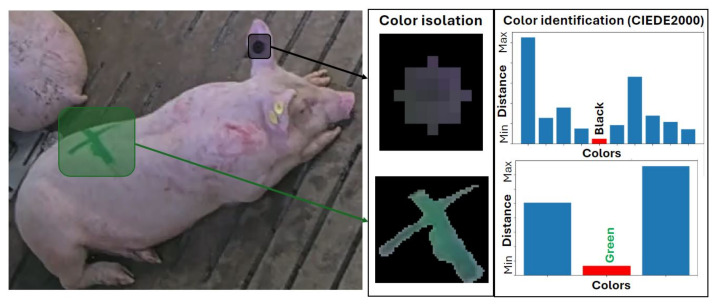
Ear tag-based and livestock mark-based identification systems.

For ear tag-based identification, a YOLOv8 object detection model [11] is used to detect the ear tag, with the left ear as input. This model was trained on 260 manually annotated ear tags, achieving an mAP50=0.991 on the test set. For the livestock mark-based identification, again, a YOLOv8 instance segmentation model [11] is used to isolate and classify the symbol (i.e., triangle, circle, line, or cross). This model was trained on 379 manually annotated symbols, with an mAP50=0.959. In both identification systems, the average RGB (Red, Green, Blue) channel values in the selected area are then converted to the LAB (Lightness, Red-Green, Yellow-Blue) color space and compared with user-defined templates, using the CIEDE2000 color difference formula [18]. The color of the ear tag or the combination of the symbol and color from the livestock mark is used to assign a unique identifier to each animal (Figure 10).

## 3. Results

This study presents a streamlined, multi-stage pipeline that leverages well-established AI models within the context of precision livestock farming. By employing YOLO-based methods for initial data preprocessing, we reduce noise and simplify the data, enabling the accurate calculation of metrics such as locomotion and body condition scores, as well as detailed behavioral analysis. This approach focuses on deployability in different scenarios, supporting parallel processing and efficient near-real-time performance without requiring architectural model modifications. In the following sections, we provide detailed evaluations of the system’s accuracy, inference times, and reliability across key metrics.

### 3.1. Dairy Cattle Health Monitoring

#### 3.1.1. Comparative Results Across Datasets

**Figure 11:**
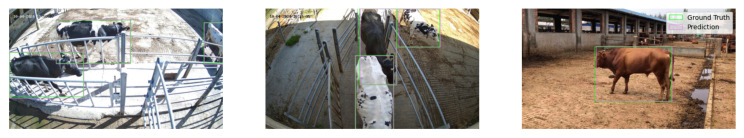
Visual comparison of different instances (with the two on the left from in-field acquisitions, and the one on the right from the NWAFU dataset [9]), with the annotated bounding boxes shown in green, and the predicted bounding boxes in purple.

**Figure 12:**
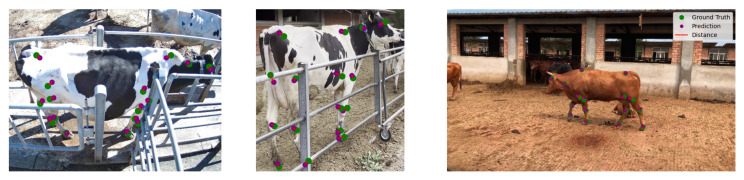
Visual comparison of different instances (with the two on the left from in-field acquisitions, and the one on the right from the NWAFU dataset [9]), with the annotated key points shown in green, the predicted key points shown in purple, and the distance between the predicted coordinates and their ground-truth values highlighted in red.

This section provides an overview of the model’s capabilities when trained on datasets collected from diverse sources. In particular, we focus on the cattle detection (Figure 11) and pose estimation models (Figure 12), highlighting their performance across various datasets. The following table (Table 2) presents a detailed comparison of the results across these datasets. The metrics shown below were computed by partitioning the test set entries by source and calculating each metric separately for each partition.

#### 3.1.2. Performance

To explore different hardware options that could be deployed on either the edge or the cloud, three different GPU node types (NVIDIA architecture) of increasing computational power were used for inference. A summary of the results in terms of accuracy and computation times on different GPU nodes is reported in Table 3. Comparable implementations on both low- and high-end computing resources indicate the applicability of scalable deep learning and edge computing solutions with adequate accuracy for efficient animal welfare monitoring. The models’ accuracies were evaluated on a validation campaign at CIRAA. A total of 34 Holstein-Friesian dairy cows were analyzed, including 29 that were previously assessed during an earlier acquisition campaign. Each animal’s ID, locomotion score (LS), and body condition score (BCS) estimated by the AI modules were compared with the annotations by trained barn personnel. The identification model correctly identified 27 of the 29 known cattle (93% accuracy). Notably, all the unseen cows were flagged as unreliable targets. With reference to the annotations by trained personnel, the mean absolute errors (MAE) were MAE(LS) = 0.28 and MAE(BCS) = 0.35.

### 3.2. Pig Behavioral Analysis

The pig identification and the posture classification systems were tested on manually annotated datasets on different NVIDIA GPU types (Table 4). The tag-based recognition system was evaluated on a set of 320 images, with Acc = 76.82%, while the mark-based system was tested on 224 images, with Acc = 85.86%. Finally, the posture classification system was compared with annotations by trained barn personnel, with Acc = 82.69%.

## 4. Discussion

We introduced a framework supporting automatic and continuous quality control procedures and observational tools for animal welfare monitoring, with applications for health status assessment and behavioral analysis. We presented two systems that can be considered already operational in experimental barn environments. The systems are based on standard video acquisition systems and a combination of pipelines of deep learning models that are considered state of the art for object detection and feature extraction. We adopted a compact implementation approach based on deep learning models that are either small or can be easily compressed into small models in terms of model size. This approach aims to build the foundation for robustness and scalability in actual PLF contexts. We note that the setups used in this work have already been tailored for operation in livestock farming without requiring an ad hoc rearrangement of spaces.

Notably, all the models we implemented have the potential for computing at the edge, that is, not necessarily involving cloud resources for inference. The small memory size footprint and the near-real-time performance observed in the computational tests with smaller-scale GPUs are encouraging in this direction.

Further, models such as the markerless identification solution can upscale to operation in very densely populated barns. Markerless identification can be difficult for dairy cow breeds with uniform skin patterns, and it could be integrated with the use of radio frequency identification devices (RFIDs). Both the locomotion score model and the BCS classifier offer a robust and automated way to estimate the health condition of animals and evaluate it over time, with the only requirement being a corridor-like structure in which cattle pass by on a regular basis. The system proposed for pig pens facilitates the assessment of interaction levels and activity within a single box (Figure 9), as well as comparisons between multiple boxes, thus representing a possible support tool for comparative studies (e.g., aggressiveness as a function of nutritional profiles). In our first experiments, both identification systems exhibited a high level of accuracy, with the livestock mark-based method proving to be more reliable compared to the ear tag-based method. This finding could be due to the smaller sizes of the ear tags and the use of standard colors that are often hard to discriminate (e.g., light blue and blue, lilac and pink). However, the ear tag-based identification method does not require periodic maintenance. The comparison with manual annotations showed the reliability of the animal posture classification system. In addition, our model for identifying and classifying interactions proved to be solid, with the limitation of not being able to robustly differentiate between frontal and central interactions. Finally, we used ByteTrack for animal tracking due to its efficient association method that accommodates low-confidence objects and its integration with YOLOv8. However, this only represents a starting point, and in the future, we plan to investigate more advanced tracking algorithms to improve the accuracy and overall performance in our analyses.

## 5. Conclusions

The two AI-based systems presented in this paper can be considered a foundational basis for the automatic assessment of key metrics of interest for monitoring the welfare and behavioral status of dairy cows and pigs on farms. The systems can minimize human effort and reduce the use of wearable devices, which are intrusive for animals. These two systems depend on opportunistic setup aspects, such as the presence of corridors and appropriate camera positioning, whose generalization could lead to an application in semi-intensive livestock systems. These systems leverage species-specific designs to address distinct welfare indicators while sharing a unified, modular architecture. Future work will focus on enhancing detection capabilities by expanding the range of indicators through integration with additional data sources or improved annotations while remaining as non-intrusive as possible. This approach aims to provide a more comprehensive evaluation of animal health and welfare across diverse farming contexts. In conclusion, this work represents a first step toward AI technologies for animal welfare monitoring, providing a set of modular solutions that can enable farmers to automate welfare assessments and take targeted actions.

## Figures and Tables

**Table 1:** List of features extracted from the pose estimation and back silhouette analysis; n1: normalized by body length; n2: normalized by the average height of hooves and elbows.

Feature	Description
Body length	Distance between withers and pin key points
Right step size	Front-back hoof distance (n1)
Left step size	Front-back hoof distance (n1)
Head position	Head height (n2)
Front-right hoof–elbow shift	Horizontal shift from front-right hoof to elbow (n1)
Front-left hoof–elbow shift	Horizontal shift from front-left hoof to elbow (n1)
Back-right hoof–elbow shift	Horizontal shift from back-right hoof to elbow (n1)
Back-left hoof–elbow shift	Horizontal shift from back-left hoof to elbow (n1)
Back straightness score	Back segmented edge line score

**Table 2:** Tabular comparison of the final model’s performance on different portions of the test set (publicly available data and in-field acquisitions) and on the complete test set.

	Public Portion		Acquired Portion		Complete Test Set	
Model	No. of Instances	mAP50	No. of Instances	mAP50	No. of Instances	mAP50
Detection	13,629	0.985	2418	0.957	16,047	0.978
Pose	3288	0.983	400	0.881	3688	0.972

**Table 3:** Performance of dairy cattle health monitoring models on test sets. Metrics are the mAP50: mean average precision at 50% intersection over union (mAP50); OKS: object key-point similarity (OKS); IoU: intersection over union (IoU); $R^{2}$; and standard accuracy. The table reports t: inference time (ms) for NVIDIA GPU types.

Model	Metric	Inference Time [ms]		
		RTX2060	RTX3060	H100
Detection	mAP50 = 0.978	37.29	34.44	26.54
Pose	mAP50 = 0.972; OKS = 0.786	20.17	19.51	10.57
Back	mAP50 = 0.951; IoU = 0.931	26.29	19.34	13.29
LS Score	$R^{2}$ = 0.96	1.93	1.48	0.15
BCS	$R^{2}$ = 0.83	22.64	18.06	10.31
Identif.	98% (Acc)	13.17	11.30	7.57

**Table 4:** Inference times (ms) for GPU types (behavioral analysis).

Model	Inference Time [ms]	
	V100	H100
Detection and Classification	25	18
Body Segmentation	27	18
Interactions	17	11
Identification (both models)	40	30
Activity Tracking	8	5

## Data Availability

The datasets analyzed in this study are not publicly available as they are part of an ongoing research project. Requests for access to the datasets can be directed to the corresponding authors.
